# Obituary

**Published:** 1877-04

**Authors:** 


### Obituary.

Dr. Heinrich Otto Gaetjens died at Denver, Col., on February 26th, 1877.

Dr. Gaetjens was born January 13th, 1838, in Hamburg, Germany, where he received a thorough collegiate education. In 1858 he went first to Wuerzburg, then to Goettingen to study medicine; at the latter university he graduated in 1863, and the following year he passed a very rigorous examination at Hamburg, to receive the appointment as assistant-surgeon to the city hospital. That position he most efficiently filled till, in 1869, he left for this country. In October, 1869, he arrived at Chicago, and took his residence on Sedgwick street, where his knowledge and skill, his kindness and unassuming manners, soon won for him the confidence of the people and the friendship of his confrères. In 1873 he participated in the founding of the German-American Dispensary. But in 1875 his health began to fail; a severe bronchitis with hæmoptysis inaugurated a rapid development of tuberculosis. He gave up the practice to seek relief and recovery, in Denver, where he died. According to his will, his remains were brought to this city, and buried in Graceland Cemetery, March 4th.

---

Gustav G. Goll died in Chicago on the morning of the 29th of March. He graduated at Rush Med. College, in 1871, and has, ever since, been a very active practitioner, striving for practice and reputation. His promising career was suddenly closed by an attack of pneumonia.

